# Supplementary material for: Toxic eburicol accumulation drives the antifungal activity of azoles against Aspergillus fumigatus
Source: Nat Commun. 2024 Jul 26;15:6312. doi: 10.1038/s41467-024-50609-1 (PMC11282106; doi:10.1038/s41467-024-50609-1)
Supplement: Supplementary file 1 — Supplementary Information [file 41467_2024_50609_MOESM1_ESM.pdf]

Toxic eburicol accumulation drives the antifungal activity of azoles against *Aspergillus fumigatus*

Supplementary Information

Elsaman, Golubtsov et al.

Supplementary Figure 1

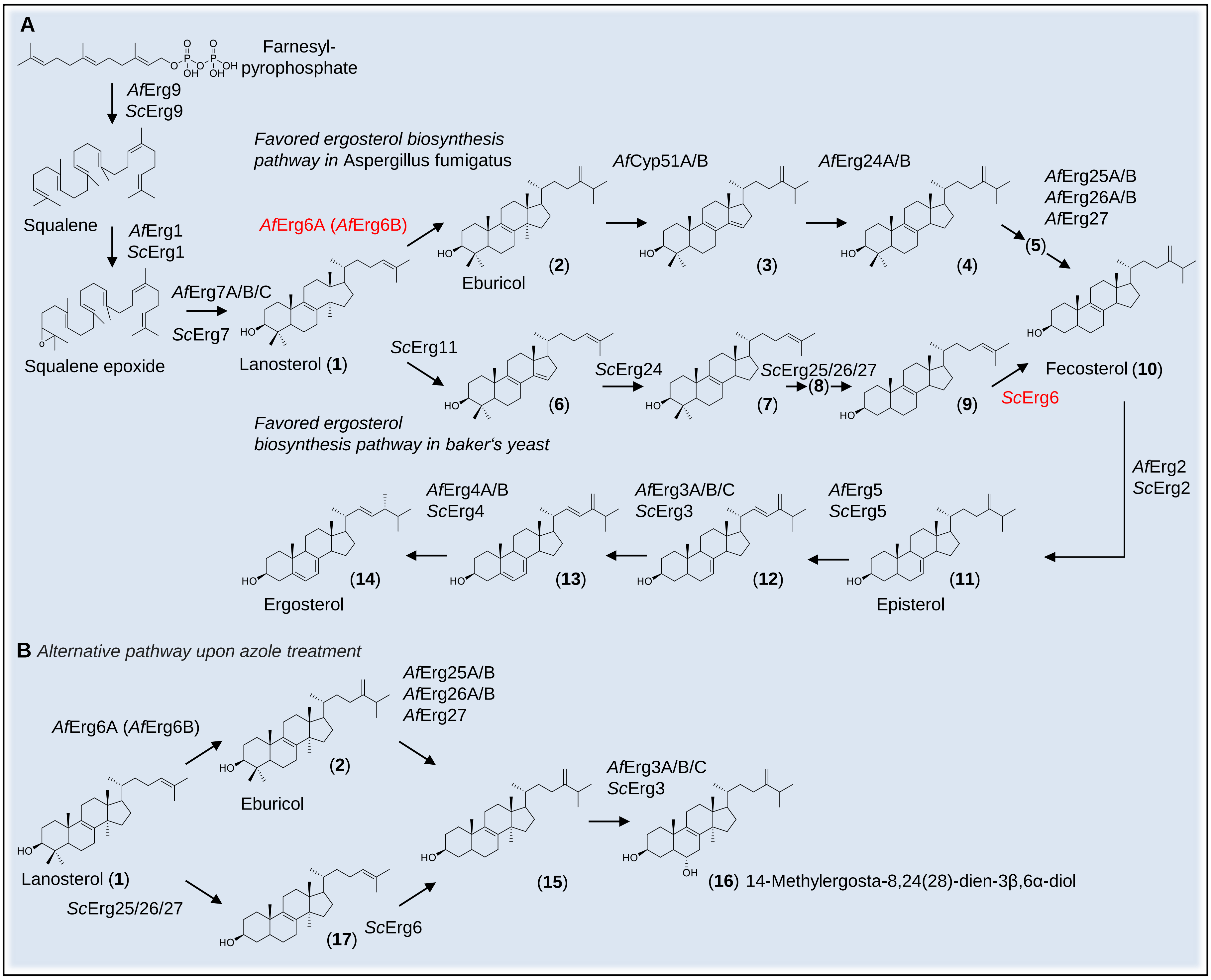

Supplementary Fig. 1. Sterol biosynthesis pathways with molecular structures of intermediates in *S. cerevisiae* and *A. fumigatus*. (A) Ergosterol biosynthesis pathways in *A. fumigatus* (*Af*; top pathway) and the baker's yeast *S. cerevisiae* (*Sc*; bottom pathway). In *A. fumigatus*, C24-methylation of lanosterol (**1**) by sterol C24-methyltransferase (*Af*Erg6A; highlighted in red) is favored over C14-demethylation. In *S. cerevisiae*, lanosterol (**1**) is the favored substrate of sterol C14-demethylase (*Sc*Erg11). Both pathways converge with the formation of fecosterol (**10**), which is then further processed to ergosterol (**14**). (B) Proposed alternative sterol biosynthesis pathway upon inhibition of sterol C14-demethylase (AfCyp51A/B and *Sc*Erg11). In *S. cerevisiae*, lanosterol (**1**) is converted to 14-methylcholesta-8,24-dien-3β-ol (**17**), which in turn is converted to 14-methylfecosterol (14-methylergosta-8,24(28)-dien-3β-ol) (**15**). In *A. fumigatus*, eburicol (**2**) is directly converted to 14-methylfecosterol (**15**). 14-methylfecosterol (**15**) is then converted to the 14-methylergosta-8,24(28)-dien-3β,6α-diol (**16**) which is considered a “toxic diol”. (A and B) Ergosterol biosynthesis enzymes in *A. fumigatus* (*Af*) and *S. cerevisiae* (*Sc*): squalene synthase (***Af*Erg9**, ***Sc*Erg9**), squalene epoxidase (***Af*Erg1**, ***Sc*Erg1**), lanosterol synthase (***Af*Erg7A/B/C**, ***Sc*Erg7**), sterol C24-methyltransferase (***Af*Erg6A**, ***Af*Erg6B** and ***Sc*Erg6**; highlighted in red), sterol C14-demethylase (***Af*Cyp51A/B**, ***Sc*Erg11**), sterol C14-reductase (***Af*Erg24A/B**, ***Sc*Erg24**), sterol C4-demethylase complex (***Af*Erg25A/B**, ***Af*Erg26A/B, *Af*Erg27**, ***Sc*Erg25, *Sc*Erg26, *Sc*Erg27**), sterol C8-isomerase (***Af*Erg2**, ***Sc*Erg2**), sterol C22-desturase (***Af*Erg5**, ***Sc*Erg5**), sterol C5-desaturase (***Af*Erg3A/B/C**, ***Sc*Erg3**), sterol C24 reductase (***Af*Erg4A/B**, ***Sc*Erg4**). Sterols: (**1**) lanosterol, (**2**) eburicol, (**3**) 4,4-dimethylergosta-8,14,24(28)-trien-3β-ol, (**4**) 4,4-dimethylergosta-8,24(28)-dien-3β-ol, (**5**) 4-methylergosta-8,24(28)-dien-3β-ol, (**6**) 4,4-dimethylcholesta-8,14,24-trien-3β-ol, (**7**) 4,4-dimethylcholesta-8,24-dien-3β-ol, (**8**) 4-methylcholesta-8,24-dien-3β-ol, (**9**) zymosterol, (**10**) fecosterol, (**11**) episterol, (**12**) ergosta-7,22,24(28)-trien-3β-ol, (**13**) ergosta-5,7,22,24(28)-tetraen-3β-ol, (**14**) ergosterol, (**15**) 14-methylfecosterol (14-methylergosta-8,24(28)-dien-3β-ol), (**16**) 14-methylergosta-8,24(28)-dien-3β,6α-diol, and (**17**) 14-methylcholesta-8,24-dien-3β-ol.

Supplementary Figure 2

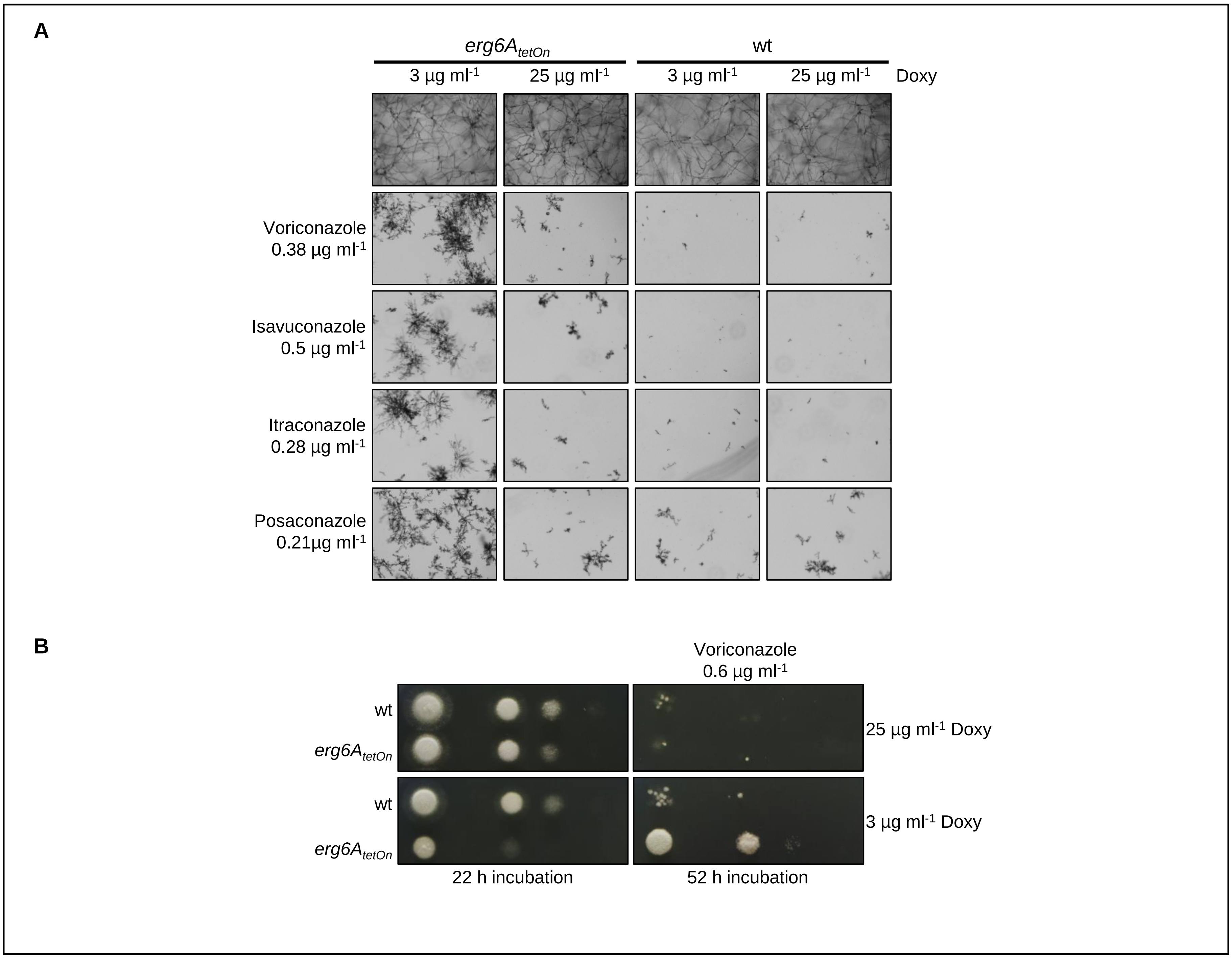

Supplementary Fig. 2. The impact of sterol C24-methyltransferase depletion on azole susceptibility. (A) Conidia of wild type and the conditional *erg6A$_{tetOn}$* mutant were inoculated in Sabouraud medium supplemented with the indicated amount of azole and doxycycline (Doxy). After 20 h incubation at 37 °C, growth was documented with an automated microscope. (B) In a series of 10-fold dilutions derived from a starting suspension of 5 × 10^7 conidia ml^{-1} of wild type (wt) and the conditional *erg6A$_{tetOn}$* mutant, aliquots of 3 µl were spotted onto Sabouraud agar plates. Medium was supplemented with the indicated amount of doxycycline or voriconazole. Agar plates were incubated at 37 °C. Representative photos were taken after the indicated incubation time.

Supplementary Figure 3

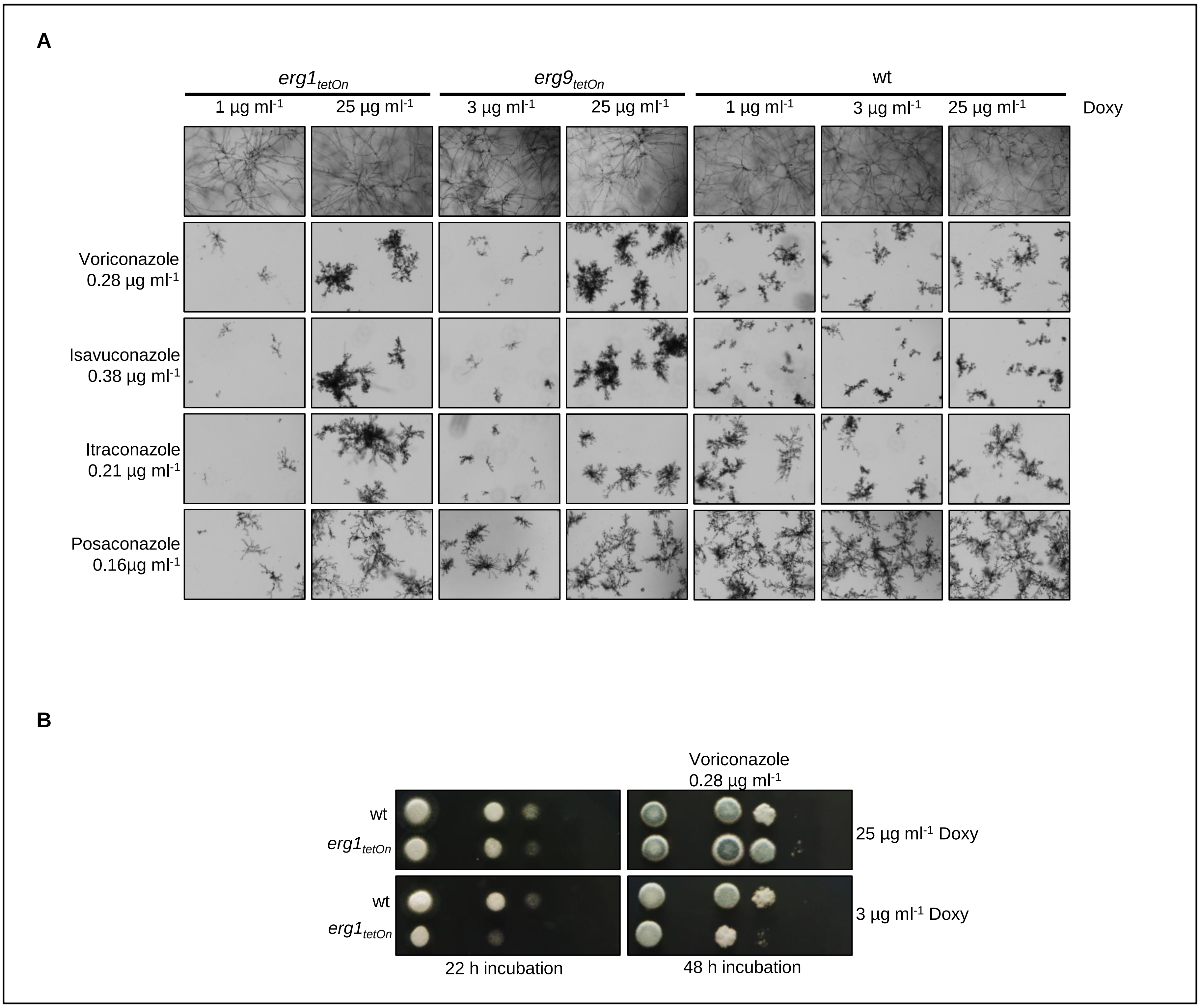

Supplementary Fig. 3. Altered expression of squalene synthase or squalene epoxidase results in changes in azole sensitivity. (A) Conidia of wild type, the conditional *erg1*$_{tetOn}$ mutant, and the conditional *erg9*$_{tetOn}$ mutant were inoculated in Sabouraud medium supplemented with the indicated amount azole and doxycycline (Doxy). After 20 h incubation at 37 °C, growth was documented with an automated microscope. (B) In a series of 10-fold dilutions derived from a starting suspension of 5 × 10^7 conidia ml^{-1} of wild type (wt) and the conditional *erg1*$_{tetOn}$ mutant, aliquots of 3 µl were spotted onto Sabouraud agar plates. Medium was supplemented with the indicated amount of doxycycline or voriconazole. Agar plates were incubated at 37 °C. Representative photos were taken after the indicated incubation time.

Supplementary Figure 4

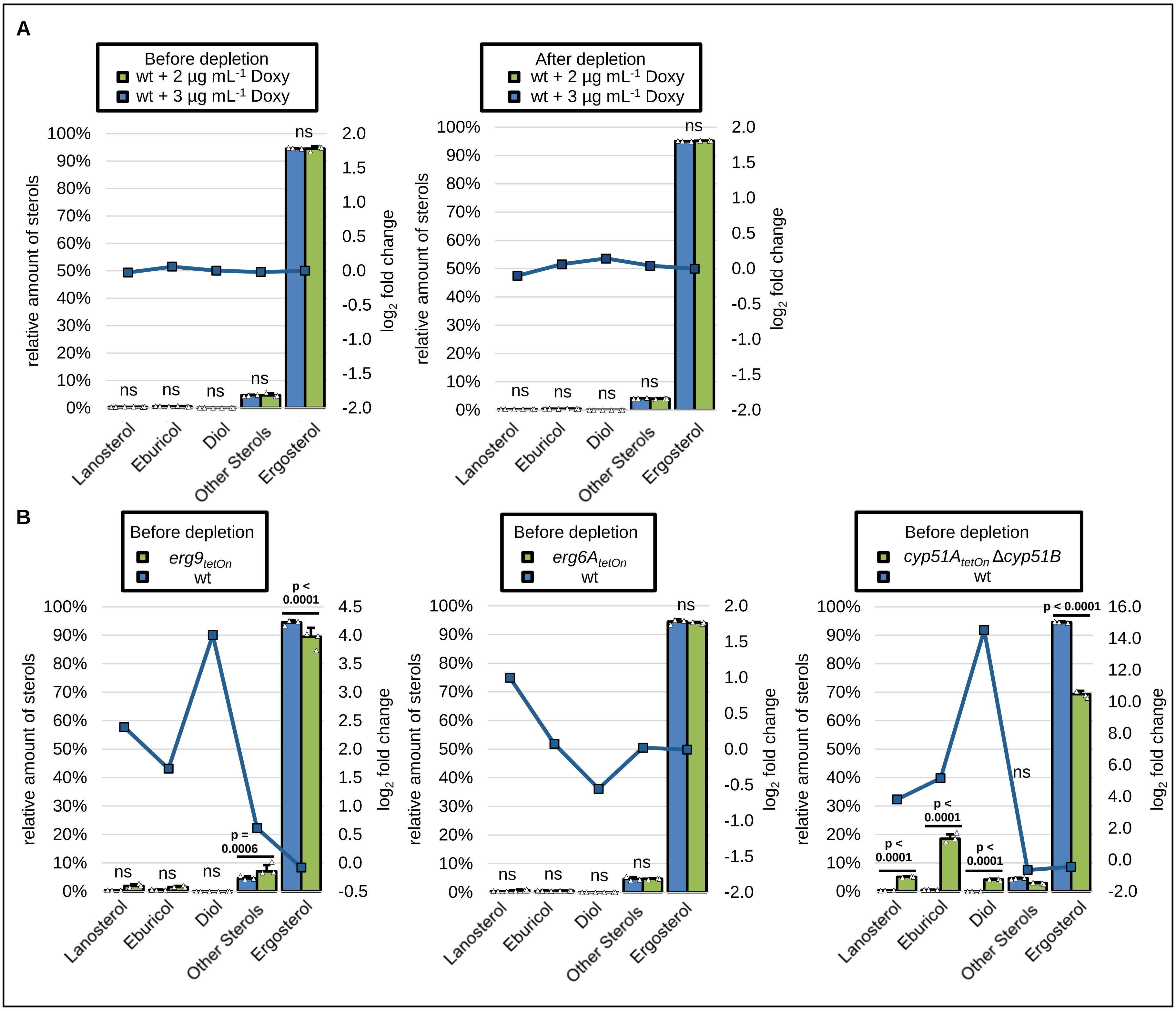

Supplementary Fig. 4. Sterol profiles of squalene synthase-, sterol C24-methyltransferase- and sterol C14-demethylase-depleted *A. fumigatus* hyphae. Further pairwise comparisons of the results of the experiment described and depicted in Fig. 4. The sterol patterns of the harvested mycelia were analyzed by gas chromatography-mass spectrometry (GC-MS). The column graphs show the relative amounts (percentage of total sterol, left y-axis) of the indicated sterols for the indicated strains before and after doxycycline depletion. The data points with the square symbols indicate the log_2-fold change (right y-axis) in the amount of the respective sterol of the pairwise comparison of the conditions shown in the individual graphs. The log_2-fold change data points were connected by lines for better visual illustration of the changes in the profiles. Each column bar represents the mean of three replicates (data points) per condition, the error bars indicate standard deviations. Data are representative of five (*erg6A*$_{tetOn}$; *cyp51A*$_{tetOn}$ Δ*cyp51B*) and one (*erg9*$_{tetOn}$) independent experiments conducted under similar conditions. Statistical significance was set at $p < 0.05$, and calculated with a two-way ANOVA with Tukey's multiple comparison test. p values are indicated in the graphs; ns, not significant. Source data are provided as a Source Data file.

Supplementary Figure 5

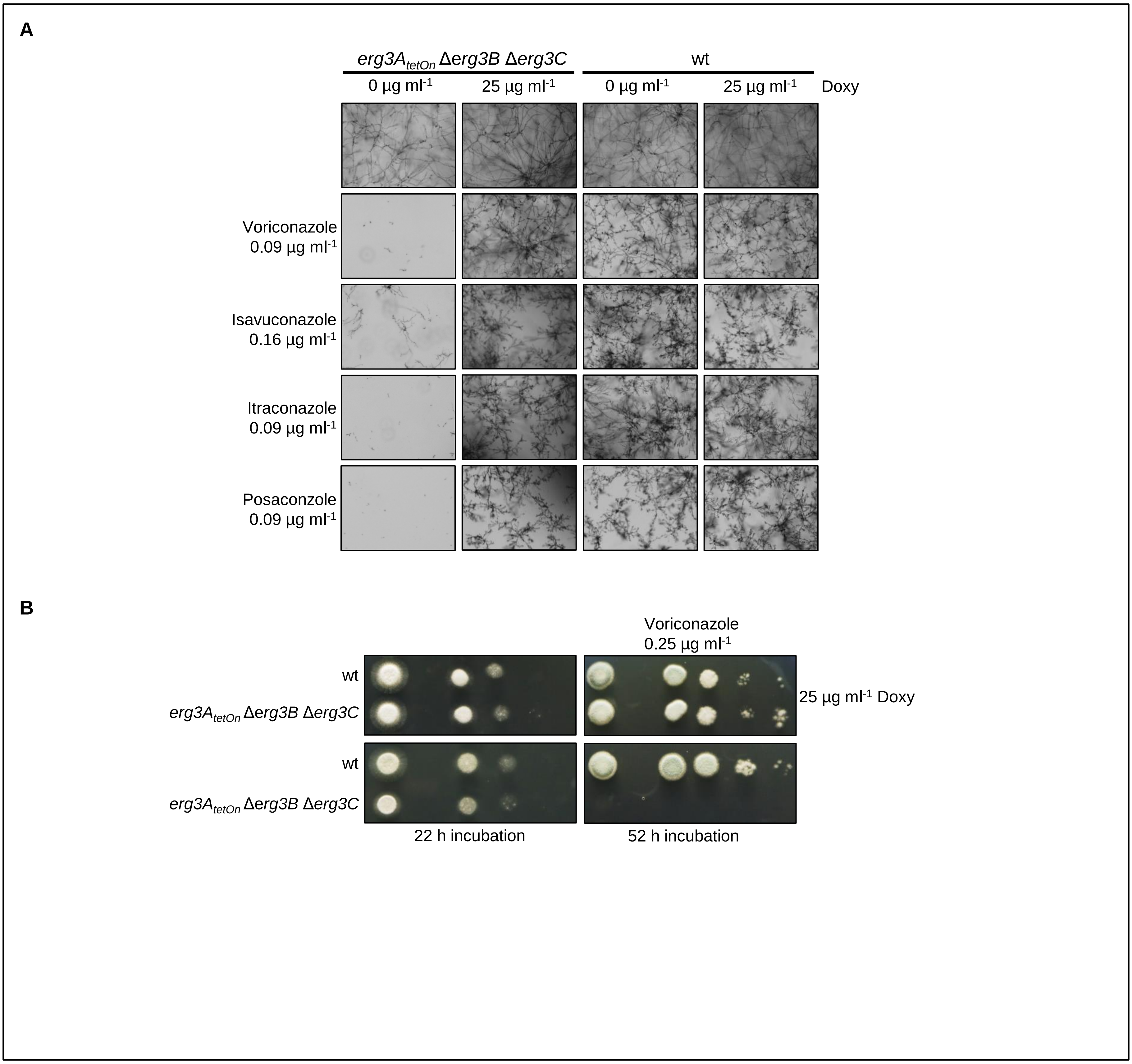

Supplementary Fig. 5. ERG3 depletion results in increased azole susceptibility. (A) Conidia of wild type and the conditional ERG3 triple mutant (*erg3A$_{tetOn}$* Δ*erg3B* Δ*erg3C*) were inoculated in Sabouraud medium supplemented with the indicated amount of azole and doxycycline (Doxy). After 20 h incubation at 37 °C, growth was documented with an automated microscope. (B) In a series of 10-fold dilutions derived from a starting suspension of 5×10^7 conidia ml^{-1} of wild type (wt) and the conditional ERG3 triple mutant (*erg3A$_{tetOn}$* Δ*erg3B* Δ*erg3C*), aliquots of 3 µl were spotted onto Sabouraud agar plates. Medium was supplemented with the indicated amount of doxycycline or voriconazole. Agar plates were incubated at 37 °C. Representative photos were taken after the indicated incubation time.

Supplementary Table 1: Sterol content of hyphae of wild type (wt) and the conditional *erg6A*$_{tetOn}$ (sterol C24-methyltransferase), *erg9*$_{tetOn}$ (squalene synthase), and *cyp51A*$_{tetOn}$ Δ*cyp51B* (sterol C14 demethylase) mutants before doxycycline depletion in %. Mean, means of three replicates per condition; SD, standard deviation. Experimental conditions are depicted in Figure 4A. Source data are provided as a Source Data file.

Doxycycline:	2µg ml^{-1}						3µg ml^{-1}			
	wt		*erg6A*$_{tetOn}$		*erg9*$_{tetOn}$		wt		*cyp51A*$_{tetOn}$ Δ*cyp51B*	
**Sterol**	**Mean**	**SD**	**Mean**	**SD**	**Mean**	**SD**	**Mean**	**SD**	**Mean**	**SD**
Ergosta-8,22,24(28)-trien-3β-ol	**0.05**	0.00	**0.05**	0.00	**0.00**	0.00	**0.06**	0.01	**0.06**	0.00
Ergosta-5,8,22-trien-3β-ol (lichesterol)	**1.15**	0.04	**1.31**	0.08	**1.08**	0.55	**1.22**	0.05	**0.62**	0.06
Ergosta-5,7,22,24(28)-tetraen-3β-ol (**13**)	**0.15**	0.01	**0.14**	0.01	**0.36**	0.09	**0.16**	0.01	**0.18**	0.01
Ergosta-X,Y-dien-3β-ol (unidentified sterol)	**0.00**	0.00	**0.00**	0.00	**0.00**	0.00	**0.00**	0.00	**0.00**	0.00
Ergosta-5,7,22-trien-3β-ol (ergosterol, **14**)	**94.46**	0.71	**94.05**	0.31	**89.38**	2.54	**94.58**	0.17	**69.27**	0.93
Ergosta-7,22-dien-3β-ol (5-dihydroergosterol)	**0.20**	0.09	**0.10**	0.02	**0.00**	0.00	**0.19**	0.01	**0.00**	0.00
14-methylergosta-8,24(28)-dien-3β-ol (14-methylfecosterol, **15**)	**0.00**	0.00	**0.00**	0.00	**0.00**	0.00	**0.00**	0.00	**0.26**	0.02
Ergosta-5,8,22,24(28)-tetraen-3β-ol	**0.26**	0.02	**0.30**	0.02	**0.42**	0.12	**0.30**	0.02	**0.26**	0.02
Ergosta-7,22,24(28)-trien-3β-ol (**12**)	**0.51**	0.35	**0.26**	0.01	**0.34**	0.18	**0.27**	0.02	**0.09**	0.01
Ergosta-5,7-dien-3β-ol	**0.61**	0.08	**0.85**	0.09	**1.02**	0.01	**0.65**	0.08	**0.51**	0.04
Ergosta-7,24(28)-dien-3β-ol (episterol, **11**)	**0.90**	0.08	**0.85**	0.02	**1.51**	0.38	**0.84**	0.04	**0.66**	0.06
14-methylergosta-8,24(28)-dien-3β,6α-diol (**16**)	**0.00**	0.00	**0.00**	0.00	**0.00**	0.00	**0.00**	0.00	**4.13**	0.22
4,4,14-trimethylcholesta-8,24-trien-3β-ol (lanosterol, **1**)	**0.37**	0.04	**0.73**	0.19	**1.85**	0.53	**0.35**	0.05	**5.07**	0.17
4-methylergosta-8,24(28)-dien-3β-ol (4-methylfecosterol, **5**)	**0.24**	0.01	**0.24**	0.01	**0.60**	0.14	**0.24**	0.02	**0.07**	0.02
4,4,14-trimetylergosta-8,24(28)-dien-3β-ol (eburicol, **2**)	**0.50**	0.13	**0.52**	0.05	**1.59**	0.31	**0.53**	0.05	**18.63**	1.26
4,4-dimethylergosta-8,24(28)-dien-3β-ol (**4**)	**0.57**	0.02	**0.54**	0.02	**1.37**	0.27	**0.59**	0.01	**0.11**	0.03
**Total sterols**	**100**		**100**		**100**		**100**		**100**	

Supplementary Table 2: Sterol content of hyphae of wild type (wt) and the conditional *erg6A*$_{tetOn}$ (sterol C24-methyltransferase), *erg9*$_{tetOn}$ (squalene synthase), and *cyp51A*$_{tetOn}$ Δ*cyp51B* (sterol C14 demethylase) mutants after doxycycline depletion in %. Mean, means of three replicates per condition; SD, standard deviation. Experimental conditions are depicted in Figure 4A. Source data are provided as a Source Data file.

Doxycycline concentration before depletion:	2µg ml^{-1}						3µg ml^{-1}			
	wt		*erg6A*$_{tetOn}$		*erg9*$_{tetOn}$		wt		*cyp51A*$_{tetOn}$ Δ*cyp51B*	
**Sterol**	**Mean**	**SD**	**Mean**	**SD**	**Mean**	**SD**	**Mean**	**SD**	**Mean**	**SD**
Ergosta-8,22,24(28)-trien-3β-ol	**0**	0.00	**0.05**	0.00	**0.05**	0.02	**0.06**	0.00	**0.02**	0.01
Ergosta-5,8,22-trien-3β-ol (lichesterol)	**1.02**	0.04	**1.06**	0.02	**1.15**	0.39	**1.01**	0.03	**0.39**	0.02
Ergosta-5,7,22,24(28)-tetraen-3β-ol (**13**)	**0.17**	0.01	**0.17**	0.01	**0.21**	0.08	**0.20**	0.01	**0.14**	0.01
Ergosta-X,Y-dien-3β-ol (unidentified sterol)	**0.00**	0.00	**0.00**	0.00	**0.00**	0.00	**0.00**	0.00	**0.00**	0.00
Ergosta-5,7,22-trien-3β-ol (ergosterol, **14**)	**95.17**	0.05	**86.99**	0.59	**89.03**	2.73	**95.06**	0.07	**41.57**	2.44
Ergosta-7,22-dien-3β-ol (5-dihydroergosterol)	**0.18**	0.01	**0.24**	0.02	**0.56**	0.11	**0.24**	0.02	**0.00**	0.00
14-methylergosta-8,24(28)-dien-3β-ol (14-methylfecosterol, **15**)	**0.00**	0.00	**0.00**	0.00	**0.00**	0.00	**0.00**	0.00	**1.17**	0.07
Ergosta-5,8,22,24(28)-tetraen-3β-ol	**0.27**	0.02	**0.31**	0.01	**0.50**	0.13	**0.31**	0.01	**0.23**	0.01
Ergosta-7,22,24(28)-trien-3β-ol (**12**)	**0.18**	0.01	**0.15**	0.01	**0.44**	0.11	**0.19**	0.01	**0.00**	0.00
Ergosta-5,7-dien-3β-ol	**0.60**	0.06	**0.56**	0.02	**1.17**	0.13	**0.63**	0.04	**0.00**	0.00
Ergosta-7,24(28)-dien-3β-ol (episterol, **11**)	**0.83**	0.02	**0.64**	0.06	**1.60**	0.18	**0.77**	0.02	**0.37**	0.02
14-methylergosta-8,24(28)-dien-3β,6α-diol (**16**)	**0.00**	0.00	**0.00**	0.00	**0.00**	0.00	**0.00**	0.00	**15.22**	0.50
4,4,14-trimethylcholesta-8,24-trien-3β-ol (lanosterol, **1**)	**0.32**	0.03	**8.38**	0.73	**1.82**	0.47	**0.30**	0.02	**10.55**	0.37
4-methylergosta-8,24(28)-dien-3β-ol (4-methylfecosterol, **5**)	**0.23**	0.00	**0.18**	0.01	**0.59**	0.15	**0.21**	0.01	**0.00**	0.00
4,4,14-trimetylergosta-8,24(28)-dien-3β-ol (eburicol, **2**)	**0.45**	0.07	**0.80**	0.12	**1.38**	0.29	**0.47**	0.01	**30.29**	1.72
4,4-dimethylergosta-8,24(28)-dien-3β-ol (**4**)	**0.50**	0.01	**0.43**	0.02	**1.38**	0.32	**0.48**	0.02	**0.00**	0.00
**Total sterols**	**100**		**100**		**100**		**100**		**100**	

Supplementary Table 3: Sterol content of hyphae of wild type (wt) and the conditional *erg3A*$_{tetOn}$ Δ*erg3B* Δ*erg3C* (C5-desaturase) mutant before and after voriconazole exposure in %. Mean, means of three replicates per condition; SD, standard deviation. Experimental conditions are depicted in Figure 5C. Source data are provided as a Source Data file.

					+ 2µg ml^{-1} voriconazole			
	wt		*erg3A*$_{tetOn}$ Δ*erg3B* Δ*erg3C*		wt		*erg3A*$_{tetOn}$ Δ*erg3B* Δ*erg3C*	
**Sterol**	**Mean**	**SD**	**Mean**	**SD**	**Mean**	**SD**	**Mean**	**SD**
Ergosta-8,22,24(28)-trien-3β-ol	**0.1**	0.01	**0.0**	0.00	**0.1**	0.00	**0.0**	0.00
Ergosta-5,8,22-trien-3β-ol (lichesterol)	**0.9**	0.05	**0.0**	0.00	**0.8**	0.01	**0.0**	0.00
Ergosta-5,7,22,24(28)-tetraen-3β-ol (**13**)	**0.2**	0.01	**0.0**	0.00	**0.2**	0.02	**0.0**	0.00
Ergosta-X,Y-dien-3β-ol (unidentified sterol)	**0.0**	0.00	**0.4**	0.02	**0.0**	0.00	**0.4**	0.03
Ergosta-5,7,22-trien-3β-ol (ergosterol, **14**)	**96.6**	0.25	**0.2**	0.11	**81.6**	0.86	**0.2**	0.13
Ergosta-7,22-dien-3β-ol (5-dihydroergosterol)	**0.1**	0.01	**8.7**	0.39	**0.3**	0.17	**10.1**	0.27
14-methylergosta-8,24(28)-dien-3β-ol (14-methylfecosterol, **15**)	**0.0**	0.00	**0.0**	0.00	**0.4**	0.03	**0.3**	0.02
Ergosta-5,8,22,24(28)-tetraen-3β-ol	**0.2**	0.02	**0.0**	0.00	**0.2**	0.01	**0.0**	0.00
Ergosta-7,22,24(28)-trien-3β-ol (**12**)	**0.2**	0.02	**74.6**	1.07	**0.7**	0.91	**65.7**	1.60
Ergosta-5,7-dien-3β-ol	**0.3**	0.07	**0.0**	0.00	**0.1**	0.01	**0.0**	0.00
Ergosta-7,24(28)-dien-3β-ol (episterol, **11**)	**0.5**	0.03	**15.3**	0.71	**0.3**	0.20	**8.3**	0.54
14-methylergosta-8,24(28)-dien-3β,6α-diol (**16**)	**0.0**	0.00	**0.0**	0.00	**2.3**	0.15	**0.0**	0.00
4,4,14-trimethylcholesta-8,24-trien-3β-ol (lanosterol, **1**)	**0.2**	0.03	**0.2**	0.01	**2.1**	0.18	**1.5**	0.13
4-methylergosta-8,24(28)-dien-3β-ol (4-methylfecosterol, **5**)	**0.1**	0.02	**0.1**	0.01	**0.0**	0.00	**0.0**	0.01
4,4,14-trimetylergosta-8,24(28)-dien-3β-ol (eburicol, **2**)	**0.2**	0.04	**0.1**	0.01	**10.8**	0.31	**13.5**	1.00
4,4-dimethylergosta-8,24(28)-dien-3β-ol (**4**)	**0.3**	0.09	**0.2**	0.02	**0.1**	0.01	**0.1**	0.02
**Total sterols**	**100**		**100**		**100**		**100**	

Supplementary Table 4: Sterol content of hyphae of wild type (wt) and the conditional *rip1*$_{tetOn}$ and *cycA*$_{tetOn}$ mutants before and after voriconazole exposure in %. Mean, means of three replicates per condition; wt, wild type; SD, standard deviation. Experimental conditions are depicted in Figure 7A. Source data are provided as a Source Data file.

							+ 1µg ml^{-1} voriconazole					
	wt		*rip1*$_{tetOn}$		*cycA*$_{tetOn}$		wt		*rip1*$_{tetOn}$		*cycA*$_{tetOn}$	
**Sterol**	**Mean**	**SD**	**Mean**	**SD**	**Mean**	**SD**	**Mean**	**SD**	**Mean**	**SD**	**Mean**	**SD**
Ergosta-8,22,24(28)-trien-3β-ol	**0.1**	0.02	**0.1**	0.03	**0.1**	0.00	**0.1**	0.00	**0.0**	0.00	**0.0**	0.00
Ergosta-5,8,22-trien-3β-ol (lichesterol)	**1.1**	0.03	**1.0**	0.03	**1.0**	0.05	**0.9**	0.10	**0.6**	0.01	**0.5**	0.03
Ergosta-5,7,22,24(28)-tetraen-3β-ol (**13**)	**0.2**	0.01	**0.2**	0.05	**0.2**	0.02	**0.2**	0.00	**0.1**	0.01	**0.1**	0.01
Ergosta-5,7,22-trien-3β-ol (ergosterol, **14**)	**95.9**	0.17	**96.5**	0.10	**96.9**	0.12	**64.7**	0.04	**46.5**	3.39	**44.9**	2.75
Ergosta-7,22-dien-3β-ol (5-dihydroergosterol)	**0.2**	0.02	**0.1**	0.01	**0.1**	0.00	**0.1**	0.01	**0.0**	0.00	**0.0**	0.00
14-methylergosta-8,24(28)-dien-3β-ol (14-methylfecosterol, **15**)	**0.0**	0.00	**0.0**	0.00	**0.0**	0.00	**0.9**	0.14	**1.5**	0.23	**1.3**	0.12
Ergosta-5,8,22,24(28)-tetraen-3β-ol	**0.3**	0.01	**0.3**	0.05	**0.3**	0.02	**0.3**	0.03	**0.3**	0.02	**0.3**	0.01
Ergosta-7,22,24(28)-trien-3β-ol (**12**)	**0.2**	0.02	**0.1**	0.02	**0.1**	0.01	**0.0**	0.00	**0.0**	0.00	**0.0**	0.00
Ergosta-5,7-dien-3β-ol	**0.4**	0.01	**0.2**	0.02	**0.2**	0.03	**0.0**	0.00	**0.0**	0.00	**0.0**	0.00
Ergosta-7,24(28)-dien-3β-ol (episterol, **11**)	**0.7**	0.05	**0.4**	0.00	**0.3**	0.04	**0.0**	0.00	**0.0**	0.00	**0.0**	0.00
14-methylergosta-8,24(28)-dien-3β,6α-diol (**16**)	**0.0**	0.00	**0.0**	0.00	**0.0**	0.00	**6.4**	0.27	**28.7**	1.89	**33.8**	2.77
4,4,14-trimethylcholesta-8,24-trien-3β-ol (lanosterol, **1**)	**0.2**	0.01	**0.2**	0.01	**0.2**	0.02	**5.8**	0.21	**6.3**	0.43	**5.6**	0.22
4-methylergosta-8,24(28)-dien-3β-ol (4-methylfecosterol, **5**)	**0.2**	0.02	**0.1**	0.02	**0.1**	0.01	**0.0**	0.00	**0.0**	0.00	**0.0**	0.00
4,4,14-trimetylergosta-8,24(28)-dien-3β-ol (eburicol, **2**)	**0.2**	0.02	**0.2**	0.01	**0.1**	0.01	**20.6**	0.48	**15.9**	0.86	**13.4**	0.71
4,4-dimethylergosta-8,24(28)-dien-3β-ol (**4**)	**0.3**	0.04	**0.3**	0.06	**0.3**	0.02	**0.0**	0.00	**0.0**	0.00	**0.0**	0.00
**Total sterols**	**100**		**100**		**100**		**100**		**100**		**100**	
