# Supplementary material for: Toxic eburicol accumulation drives the antifungal activity of azoles against Aspergillus fumigatus
Source: Nat Commun. 2024 Jul 26;15:6312. doi: 10.1038/s41467-024-50609-1 (PMC11282106; doi:10.1038/s41467-024-50609-1)
Supplement: Supplementary file 3 — Reporting Summary [file 41467_2024_50609_MOESM3_ESM.pdf]

nature portfolio

Corresponding author(s): Johannes Wagener

Last updated by author(s): Jun 22, 2024

Reporting Summary

Nature Portfolio wishes to improve the reproducibility of the work that we publish. This form provides structure for consistency and transparency in reporting. For further information on Nature Portfolio policies, see our Editorial Policies and the Editorial Policy Checklist.

Statistics

For all statistical analyses, confirm that the following items are present in the figure legend, table legend, main text, or Methods section.

n/a	Confirmed	
☐	☒	The exact sample size (n) for each experimental group/condition, given as a discrete number and unit of measurement
☐	☒	A statement on whether measurements were taken from distinct samples or whether the same sample was measured repeatedly
☐	☒	The statistical test(s) used AND whether they are one- or two-sided<br>*Only common tests should be described solely by name; describe more complex techniques in the Methods section.*
☒	☐	A description of all covariates tested
☐	☒	A description of any assumptions or corrections, such as tests of normality and adjustment for multiple comparisons
☐	☒	A full description of the statistical parameters including central tendency (e.g. means) or other basic estimates (e.g. regression coefficient) AND variation (e.g. standard deviation) or associated estimates of uncertainty (e.g. confidence intervals)
☐	☒	For null hypothesis testing, the test statistic (e.g. F, t, r) with confidence intervals, effect sizes, degrees of freedom and P value noted<br>*Give P values as exact values whenever suitable.*
☒	☐	For Bayesian analysis, information on the choice of priors and Markov chain Monte Carlo settings
☒	☐	For hierarchical and complex designs, identification of the appropriate level for tests and full reporting of outcomes
☒	☐	Estimates of effect sizes (e.g. Cohen's d, Pearson's r), indicating how they were calculated

Our web collection on statistics for biologists contains articles on many of the points above.

Software and code

Policy information about availability of computer code

Data collection	not applicable
Data analysis	GraphPad Prism (Version 10.1.1 (Prism 10); Dotomatics, Boston, MA, USA)<br>Excel, Microsoft Office 365

For manuscripts utilizing custom algorithms or software that are central to the research but not yet described in published literature, software must be made available to editors and reviewers. We strongly encourage code deposition in a community repository (e.g. GitHub). See the Nature Portfolio guidelines for submitting code & software for further information.

Data

Policy information about availability of data

All manuscripts must include a data availability statement. This statement should provide the following information, where applicable:

- Accession codes, unique identifiers, or web links for publicly available datasets
- A description of any restrictions on data availability
- For clinical datasets or third party data, please ensure that the statement adheres to our policy

The data that support the findings of this study are included in this article and its Supplementary Information files, in the Source Data file or on request from the corresponding author.

Research involving human participants, their data, or biological material

Policy information about studies with human participants or human data. See also policy information about sex, gender (identity/presentation), and sexual orientation and race, ethnicity and racism.

Reporting on sex and gender	not applicable
Reporting on race, ethnicity, or other socially relevant groupings	not applicable
Population characteristics	not applicable
Recruitment	not applicable
Ethics oversight	not applicable

Note that full information on the approval of the study protocol must also be provided in the manuscript.

Field-specific reporting

Please select the one below that is the best fit for your research. If you are not sure, read the appropriate sections before making your selection.

☒ Life sciences ☐ Behavioural & social sciences ☐ Ecological, evolutionary & environmental sciences

For a reference copy of the document with all sections, see nature.com/documents/nr-reporting-summary-flat.pdf

Life sciences study design

All studies must disclose on these points even when the disclosure is negative.

Sample size	The sample sizes were not calculated. The sample sizes for the sterol analyses, which constituted the only experiments with statistical analyses, were chosen based on the recommended optimal sample size (three replicates; Nat Protoc. 2017 May;12(5):947-963. doi: 10.1038/nprot.2017.005). For all other experiments, the sample size was selected according to what appeared technically feasible and comprised at least three replicates.
Data exclusions	No data were excluded from analyses. The traces of cholesterol detected in the sterol analyses was considered a contamination from the growth medium and are not listed in the supplementary tables.
Replication	Experiments were generally performed at least three times under similar conditions with consistent results with the exception of the sterol analyses of the conditional ERG3 mutant, which was conducted three times but with two independent ERG3 triple mutants (with consistent results, one mutant per experiment), and of the conditional mitochondrial complex III mutants which was conducted once but with two independent mutants (cycA and rip1) with consistant results.
Randomization	Randomization was not performed. The work was carried out with a defined number of strains that were directly compared with each other.
Blinding	Blinding was not performed because either the samples/mutant could be identified by the assessor due to obvious qualitative differences (i.e., in growth rates or in presence/absence of patches) or the analysis of the samples was performed using a technical method (sterol analyses) in which the measurements could not be influenced by the assessor.

Reporting for specific materials, systems and methods

We require information from authors about some types of materials, experimental systems and methods used in many studies. Here, indicate whether each material, system or method listed is relevant to your study. If you are not sure if a list item applies to your research, read the appropriate section before selecting a response.

Materials & experimental systems

n/a	Involved in the study	
☒	☐	Antibodies
☒	☐	Eukaryotic cell lines
☒	☐	Palaeontology and archaeology
☒	☐	Animals and other organisms
☒	☐	Clinical data
☐	☒	Dual use research of concern
☒	☐	Plants

Methods

n/a	Involved in the study	
☒	☐	ChIP-seq
☒	☐	Flow cytometry
☒	☐	MRI-based neuroimaging

Dual use research of concern

Policy information about dual use research of concern

Hazards

Could the accidental, deliberate or reckless misuse of agents or technologies generated in the work, or the application of information presented in the manuscript, pose a threat to:

No	Yes	
☒	☐	Public health
☒	☐	National security
☒	☐	Crops and/or livestock
☒	☐	Ecosystems
☒	☐	Any other significant area

Experiments of concern

Does the work involve any of these experiments of concern:

No	Yes	
☒	☐	Demonstrate how to render a vaccine ineffective
☐	☒	Confer resistance to therapeutically useful antibiotics or antiviral agents
☒	☐	Enhance the virulence of a pathogen or render a nonpathogen virulent
☒	☐	Increase transmissibility of a pathogen
☒	☐	Alter the host range of a pathogen
☒	☐	Enable evasion of diagnostic/detection modalities
☒	☐	Enable the weaponization of a biological agent or toxin
☒	☐	Any other potentially harmful combination of experiments and agents

Precautions and benefits

Biosecurity precautions	The work was conducted in BSL2 labs with controlled access with appropriate control measures on place.
Biosecurity oversight	The work was carried out with the approval of the governments of Lower Franconia and Upper Bavaria (Bavaria, Germany) and the Environmental Protection Agency (Ireland).
Benefits	The work conducted contributes to a better understanding of the development of antifungal resistance and to a better understanding of the mode of action of antifungal agents used to treat fungal infections.
Communication benefits	The benefits of understanding the mode of action of azole antifungals and the potentially associated mechanisms of resistance far outweigh the little-to-no risk that the findings could pose a risk to public health or national security as such resistance can occur naturally upon use of the antifungal drugs.

Plants

Seed stocks	not applicable
Novel plant genotypes	not applicable
Authentication	not applicable
